# Expanding the experimental EuroQol toddler and infant populations (EQ-TIPS) descriptive system: comparing 3-level and 5-level performance for infants and toddlers with chronic conditions

**DOI:** 10.1186/s41687-026-01113-1

**Published:** 2026-06-12

**Authors:** Janine Verstraete, Marco Zampoli, Alan Davidson, Marc Hendricks, Helder de Quintal, Yasmin Goga, Jo M. Wilmshurst, Alvin Ndondo, Gill Riordan, Ronalda De Lacy, Mignon McCulloch, Deveshni Reddy, Lasse Herdien

**Affiliations:** 1 Department of Paediatrics and Child Health, Faculty of Health Sciences, University of Cape Town, Cape Town, South Africa; 2 Division of Pulmonology, Department of Paediatrics and Child Health, Faculty of Health Sciences, University of Cape Town, Cape Town, South Africa; 3 Red Cross War Memorial Children’s Hospital, Cape Town, South Africa; 4 Division of Haematology and Oncology, Department of Paediatrics and Child Health, Faculty of Health Sciences, University of Cape Town, Cape Town, South Africa; 5 Department of Paediatric Neurology, Red Cross War Memorial Children’s Hospital, Cape Town, South Africa; 6 Neuroscience Institute, University of Cape Town, Cape Town, South Africa; 7 Division of Gastroenterology, Department of Paediatrics and Child Health, Faculty of Health Sciences, University of Cape Town, Cape Town, South Africa; 8 Division of Nephrology, Department of Paediatrics and Child Health, Faculty of Health Sciences, University of Cape Town, Cape Town, South Africa

**Keywords:** Health related quality of life, Outcome measure, Infant, Toddler, Child, EQ-TIPS

## Abstract

**Objectives:**

The EuroQol Infant and Toddlers Populations (EQ-TIPS) instrument may support both 3-level and 5-level versions, mirroring the structure of the EuroQol Adult and Youth descriptive systems. This study compared the performance of the experimental version 2.0 EQ-TIPS-3L (3 L) with EQ-TIPS-5L (5L) in children 0–4 years living with a health condition.

**Methods:**

Caregivers of children were recruited from specialist outpatient clinics in South Africa. The feasibility of EQ-TIPS-5L and EQ-TIPS-3L was compared using the absolute reduction in the ceiling effect (111111). Discriminatory power was evaluated with Shannon’s H’ and J’ index for absolute and relative informativity. The redistribution of dimension responses between the 5 L and 3L versions was evaluated for inconsistency, defined as a 5 L response that was two or more levels removed from a 3 L response. Convergent validity of EQ-TIPS dimensions was calculated with Kendall Tau B and Gamma correlations. Known-group severity was calculated for the EQ-TIPS-5 L and EQ-TIPS-3 L LSS scores using severity groupings based on PedsQL total score (≤ 74.2) and EQ VAS (≤ 80).

**Results:**

Data from 176 children with a median age of 29 months, and more males (54%) were included. Most respondents were mothers (78%). Children were grouped by disease category: epilepsy (9%), neuromuscular disease (7%), chronic gastrointestinal disease (16%), renal disease (27%) and oncological or haematological diseases (41%). The ceiling effect (111111) decreased minimally by 2% points from the 3L to 5L. Absolute informativity (H’) of dimensions improved by 0.096 on the 5L, with retention of the spread of responses for all dimensions. Convergent validity was similar, with strong correlations on paired 3L and 5L dimensions. The 3L and 5L were similarly able to significantly differentiate between known groups based on PedsQL and EQ VAS severity cut-offs.

**Conclusion:**

These results indicate that a 5L version of the EQ-TIPS does not influence the ceiling effect in the same way as the Adult and Youth descriptive system. The modest increase in informativity of the EQ-TIPS-5L suggests the potential advantages of expanded levels of reporting, but this requires testing in samples with more frequent reporting of severe health states.

**Supplementary Information:**

The online version contains supplementary material available at https://doi.org/10.1186/s41687-026-01113-1.

## Background

Historically, healthcare for young children has heavily emphasised reducing mortality rates [1]. With significant advances in medicine and public health, the survival rates of children have improved dramatically [2]. This positive shift necessitates a broader focus within paediatric care. As mortality rates decline, the impact of morbidity—the burden of disease—becomes increasingly crucial. This is particularly relevant in early childhood, a period of rapid neurological development [3]. The concept of health-related quality of life (HRQoL) offers a comprehensive approach to assessing children, considering not only the presence of disease but also its impact on various aspects of their lives. Impaired HRQoL during childhood can have long-lasting consequences. Neurological development during these early years lays the foundation for future cognitive, emotional, and social functioning. Disruptions in this process can lead to challenges in areas such as scholastic achievement, attention, and overall life performance [3–5]. By recognising the importance of HRQoL and its potential long-term impact, healthcare providers can adopt a more holistic approach to paediatric care [6]. This shift in perspective has implications for the future outcomes and potentially the associated health costs of these children.

Globally, the leading causes of mortality in children under five years of age are attributed to infectious diseases, including pneumonia, diarrhoea and malaria, with ongoing efforts, particularly in low-income countries, to reduce the incidence of communicable diseases [7]. Health care in high-income countries is largely focused on non-communicable diseases. The number of children living with disabilities, including long-term physical, mental, intellectual or sensory impairments, is estimated at 240 million [8]. This includes children with genetic conditions, perinatal complications and/or nutritional deficiency or infection resulting in long-term functional consequences [7]. There is further recognition that behaviours (e.g., diet and physical activity) and environmental exposures in early life impact approximately 70% of premature deaths in adults [7]. Measuring HRQoL allows healthcare professionals to assess the burden of these conditions on children and their families, tailor interventions to individual needs, and monitor the effectiveness of treatments over time. Moreover, incorporating HRQoL into economic evaluations enables policymakers to make informed decisions about resource allocation and prioritise interventions that offer the greatest value for money in improving children’s health outcomes.

The growing need for comprehensive assessment tools to evaluate HRQoL in young children is being met with the increasing number of generic multi-attribute utility instruments [9]. Most of these instruments have been developed for children older than 5 years. Currently, four measures for children younger than 5 years have been developed for proxy completion and have utility values or are amenable to eliciting preference-weighted values [9]. These instruments include the Infant Health-Related Quality of Life Instrument (IQI) [10], Health Utilities Preschool for children (HuPS) [11, 12], EuroQol Infant and Toddlers Populations (EQ-TIPS) [13, 14], and potentially the Pediatric Quality of Life Inventory (PedsQL) Generic Measures for infants and toddlers [15]. The IQI measures HRQoL in infants aged 0–12 months and does not currently have versions for toddlers or older children [10]. The HuPS is recommended for children aged 2–5 years and, together with the Health Utilities Index Mark 3 (HUI 3), allows measurement across childhood and adulthood [11]. The PedsQL is a widely used generic health assessment tool, with versions available for infants (1–12 months and 13–24 months), young children (2–4 years and 5–7 years), children (8–12 years), and adolescents (13–18 years) [15, 16]. There are ongoing efforts to develop a PedsQL classification system, which will be amenable to generating preference-weighted scores [17, 18]. The EQ-TIPS was originally developed for 0–3 years [14], with more recent research expanding this to 0–4 years [19, 20], and forms part of the EuroQol family of instruments, which includes youth (EQ-5D-Y-3L and EQ-5D-Y-5L) and adult versions (EQ-5D-3L and EQ-5D-5L) [21]. The EuroQol measurement system is currently uniquely positioned as it is the only family of measures to allow measurement and valuation of health across the lifespan from birth to adulthood.

Although there are currently no Health Technology Assessment (HTA) guidelines for infants and toddlers [22, 23], the youth version, EQ-5D-Y, is recommended by the Dutch Healthcare Institute (ZIN) and the adult version, EQ-5D-5L, is the most commonly cited preference-based measure in guidelines across the globe [24]. The EQ-5D adult and youth instruments include versions with 3 and 5-level response scales [25]. The 5-level version is thought to reduce the ceiling effect (where respondents cluster at the highest level of functioning) and increase the instrument’s sensitivity to changes in health status [26, 27]. Although concerns were noted that increasing from three to five response options would increase cognitive burden, this does not seem to be the case for adult or child respondents from high-literacy regions [26, 27]. However, EQ-TIPS experimental version 2 (EV2.0) utilises a 3-level response scale. The EQ-TIPS may support both 3-level and 5-level versions, mirroring the structure of the EQ-5D Adult and Youth descriptive systems and potentially allowing for greater consistency and easier transition between the instruments. Since EQ-TIPS is designed for proxy completion, it is essential to investigate whether the 5-level version offers any advantages over the 3-level version. This study therefore aimed to compare the performance of the EQ-TIPS-3L (3L) and the EQ-TIPS-5L (5L) in infants and toddlers aged 0–4 years living with a chronic health condition.

## Methods

### Study design and setting

A cross-sectional study was conducted among caregivers of children with chronic health conditions aged 0–4 years. Participants consisted of caregivers, typically a parent, of infants and toddlers attending a tertiary paediatric hospital in the Western Cape, South Africa. Red Cross War Memorial Children’s Hospital treats over 250,000 patients a year in both the acute and chronic services. Children with the following diagnoses were included: Oncological or Haematological Disease; Epilepsy; Neuromuscular Disease; Gastrointestinal Disease, including Liver Disease and/or transplantation; Renal Disease, including dialysis and transplantation. These diagnoses were hypothesised to result in problems across the EQ-TIPS descriptive system and levels of severity. Caregivers and their children were recruited from the dedicated specialist clinics, where parents were unable to report on the specific diagnosis the health records were consulted.

### Instruments

#### EuroQol toddler and infant population (EQ-TIPS) experimental version 2.0

The EQ-TIPS is an experimental measure developed by the EuroQol Group to measure and value HRQoL in infants and toddlers. EuroQol experimental measures are tools which are still under development and may change as development proceeds [28]. The EQ-TIPS is a proxy-report instrument completed by the primary caregiver, typically the parent.

The EQ-TIPS EV 2.0 descriptive system comprises six dimensions: Movement, Play, Pain, Communication, Social Interaction, and Eating. On the three-level version of EQ-TIPS, each dimension has 3 levels of severity corresponding to: “no problems”, “some problems” and “a lot of problems”. The EQ-TIPS-5L (5L) has 5 levels of severity corresponding to: “no problems”, “a little bit of a problem”, “some problems”, “a lot of problems”, and “extreme problems”. The response on each dimension is defined as a 1-digit number that expresses the level selected for that dimension, where “no problems” is assigned a ‘1’ and the most extreme level a ‘3’ or a ‘5’, depending on whether the 3-level or 5-level version is used. The digits for each of the six dimensions can be combined into a 6-digit number that describes the younger patient’s health state. For example, on the EQ-TIPS 5-level version, the health state 111245 would represent “no problems” on the dimensions of Movement, Play, and Pain, “a little bit of a problem” with Social Interaction, “a lot of problems” with Communication, and “extreme problems” with Eating.

The EQ-TIPS does not yet have a preference-based score; therefore, a level sum score (LSS), similar to that used on the EQ-5D [29], was used to describe the responses on the descriptive system where the level labels are treated as numeric data with the EQ-TIPS-3L and EQ-TIPS-5L scores ranging between 6-18 and 6–30, respectively. The EQ-TIPS was designed to be amenable to the development of preference weights in the future.

EQ VAS is used to record the proxy’s view of how good or bad the infant’s health is overall on the day of questionnaire completion. The EQ VAS is a vertical visual analogue scale with endpoints labelled “the best health you can imagine” and “the worst health you can imagine”.

While the EQ-5D-Y-3L and EQ-5D-Y-5L are recommended for proxy completion between 4 and 18 years [25]. The EQ-TIPS dimensions are considered more appropriate and have been shown to perform better than the EQ-5D-Y-3L in 4-year-old South African children [19, 30]. Thus, the EQ-TIPS was used for children 0–4 years in this study.

#### Study procedure

Ethical approval was obtained from the University Human Research and Ethics Committee (HREC 740_2021). As the EQ-TIPS-5L was newly developed and required testing in the source language before translation, only caregivers who could complete the surveys in English were included. Caregivers of children receiving outpatient health care were recruited from the waiting rooms of specialist clinics at the children’s hospital. Each caregiver agreed to participate in the study and provided written consent. Data from caregivers were collected between 1 December 2023 and 30 November 2024, on handheld electronic devices and managed using REDCap electronic data capture tools hosted at the University of Cape Town [31]. The researcher assisted with electronic completion, if needed, and/or was available for any clarification. The survey included information regarding the study and informed consent, EQ-TIPS-3L, PedsQL generic module, demographic and medical information for the child, EQ-TIPS-5L, EQ VAS and caregiver HRQoL and demographic information. The order of the EQ-TIPS-3L and EQ-TIPS-5L was randomised and always separated by the age-specific version of the PedsQL (0–12 months, 13–24 months or 2–4 years).

### Statistical analysis

The analysis of the 3L and 5L followed that of Janssen et al. (2008) for comparison of the adult versions EQ-5D-3L and EQ-5D-5L [32]. Randomisation was tested as a covariate for all analyses. All data analyses were conducted using SPSS Windows 29.0 (IBM SPSS Inc., Chicago, IL, USA).

#### General performance

The EQ-TIPS and descriptive data were summarised as frequencies and Median (IQR). The ceiling of the EQ-TIPS was defined as the proportion scoring “no problems” across all six dimensions (111111) and for each individual dimension. The absolute reduction in proportion from the 3L to the 5L was calculated (ceiling_Y−3 L_- ceiling_Y−5 L_).

#### Redistribution EQ-TIPS-3L responses to the EQ-TIPS-5L

**Fig. 1:**
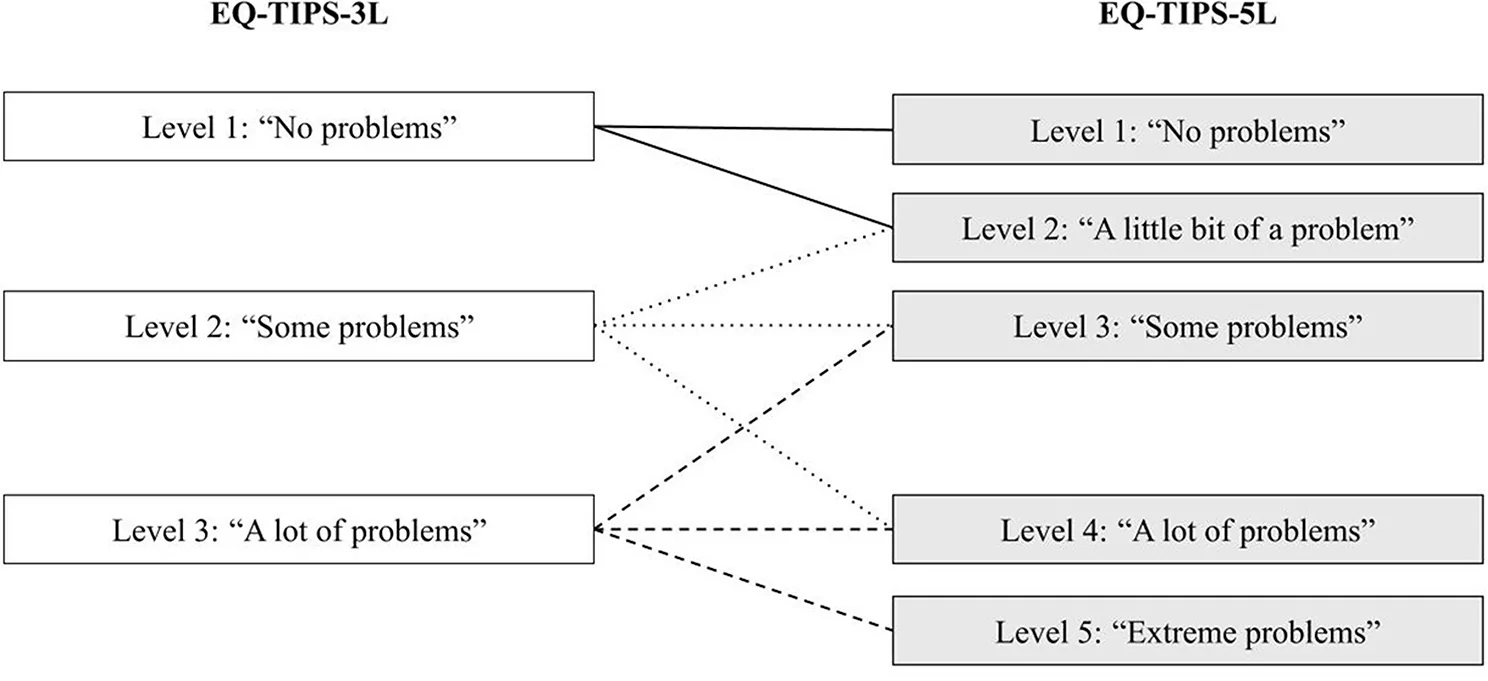
Anticipated redistribution of EQ-TIPS-3L responses to the EQ-TIPS-5L

The redistribution of paired-dimension responses was tabulated and plotted using Sankey diagrams. Paired dimension responses on the 3L and 5L were assessed for inconsistency using criteria established in previous studies comparing the adult EQ-5D versions [32, 33]. A response pair was considered inconsistent if the 5L response was at least two levels away from the 3L response (see Fig. 1).

#### Discriminatory power

The Shannon Index (H’) and the Shannon Evenness Index (J’) were used to evaluate the discriminatory power of the 3L and 5L dimensions in terms of absolute and relative informativity [32, 34]. The Shannon H’ and J’ indices are defined as follows:

$$\:{H}^{{\prime\:}}=-{\sum\:}_{i=1}^{L}{p}_{i}{log}_{2}{p}_{i}\:\mathrm{a}\mathrm{n}\mathrm{d}\:{J}^{{\prime\:}}=\frac{H{\prime\:}}{{H{\prime\:}}_{max}}$$

Where H’ is the absolute amount of informativity, L is the number of dimensions levels and p_*i*_ is the proportion of observations in the in the *i*^th^ level where 3L has three levels and 5L has five levels. A higher H’ index reflects that the descriptive system has captured more information; the maximum H’ index is 1.58 and 2.32 on the 3L and 5L, respectively. It was expected that the H’ index would be higher for the EQ-TIPS-5L compared to the EQ-TIPS-3L. The Shannon Evenness Index (J’) reveals the distribution of responses across all levels, irrespective of the total number of levels within the descriptive system. It was expected that the J’ index would be similar for the EQ-TIPS-5L and the EQ-TIPS-3L.

#### Convergent validity

The convergent validity between 3L and 5L was evaluated by dimension response pairs, using Kendall tau B and Gamma statistic. Correlation coefficients were interpreted according to Cohen: 0.1–0.29 low association, 0.3–0.49 moderate association and ≥ 0.5 high association [35]. A strong correlation was expected when examining comparable dimensions across these instruments.

#### Known group analysis by severity groups

Considering the heterogeneous nature of this data, clinically relevant known group analysis was not possible. Known groups were defined by a cut-off score for chronically ill children for the PedsQL (≤ 74.2) taken from Varni 2003 [36] and an EQ VAS of ≤ 80 [37]. These scores have previously been applied in known group severity analysis in psychometric studies [38, 39]. The EQ-TIPS-3L and EQ-TIPS-5L LSS scores were compared with the Mann-Whitney U-test for groups dichotomised by these PedsQL and EQ VAS scores.

## Results

**Fig. 2:**
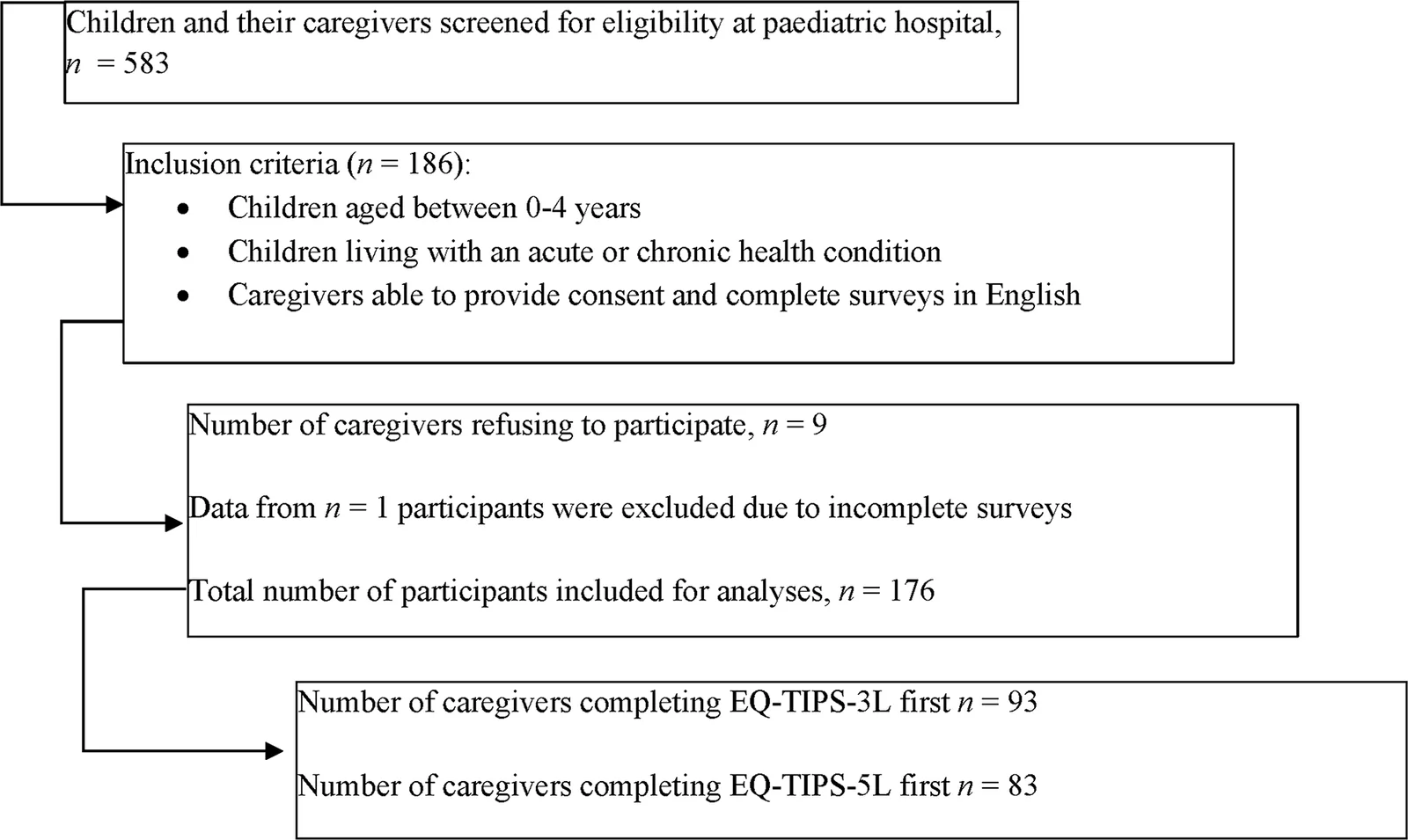
Flow diagram outlining the participant recruitment process

A total of 583 children were screened for eligibility in the specialist clinics; 397 of these were not in the EQ-TIPS age range and were recruited into a study using the EQ-5D-Y-5L (reported separately) (Fig. 2). Data from 176 caregivers with young children (median age of 29 months) were included (see Fig. 2). The 3L was completed first by 93 (52%) caregivers, whereas the 5L was completed first by 83 (48%) caregivers (*z* = 1.066, *p* = 0.286) (Fig. 2). Randomisation was well distributed across disease groups (*X*^2^ = 6.184, *p* = 0.186) with 3L completed first about half the group (Oncology (*n* = 36, 50%), Renal (*n* = 30, 63%), Gastrointestinal Disease (*n* = 13, 46%), Neuromuscular Disease (*n* = 6, 46%), and Epilepsy (*n* = 8, 53%)). Similarly, randomisation was well distributed across age groups (*X*^2^ = 0.404, *p* = 0.817) with 3L completed first in about half the group (0–12 months (*n* = 19, 54%), 13–24 months (*n* = 21, 57%), and 2–4 years (*n* = 53, 51%).

Although not all caregivers were home language English speakers, they all felt comfortable completing the survey in English. Thus, no one was excluded based on their inability to complete the survey in English. Most children were males (54%), and the most prevalent health condition was Haematological/Oncology (see Table 1). The majority of caregivers were mothers (78%), who were full-time (34%) or unemployed but looking for work (50%), and the highest completed education level was high school (51%).

The overall ceiling effect (111111) was minimally reduced moving from the 3L to the 5L version (-2% points). There was a reduction in reporting of “no problems” for Movement, Play and Pain. There was no floor effect for the EQ-TIPS on either version, but a reduction in reporting the most severe level was seen for all dimensions on the 5L version (see Table 2). Randomisation was tested as a covariate and found to be non-significant.

**Table 1:** Participant and caregiver characteristics

		Total (*N* = 176)
**Characteristics of the Child**		
Age	Median (IQR)	29 (13.5, 48.0)
Age Group	0–12 months, n (%)	35 (20)
	13–24 months, n (%)	37 (21)
	2–4 years, n (%)	104 (59)
Gender	Female, n (%)	80 (45)
	Male, n (%)	95 (54)
	Missing, n (%)	1 (1)
Disease Category	Haematological/Oncology, n (%)	72 (41)
	Renal Disease*, n (%)	48 (27)
	Chronic Gastrointestinal Disease^#^, n (%)	28 (16)
	Neuromuscular Disease, n (%)	13 (7)
	Epilepsy, n (%)	15 (9)
EQ-TIPS	VAS, Best health (100), n (%)	64 (36)
	VAS, Median (IQR)	90 (73,100)
	VAS ≤ 80, n (%)	61 (35)
	VAS > 80, n (%)	113 (64)
	3 L LSS, Median (IQR)	6 (6,8)
	5 L LSS, Median (IQR)	6 (6,9)
PedsQL	Total, Median (IQR)	86 (75,93)
	Total Score ≤ 74.2, n (%)	44 (25)
	Total Score > 74.2, n (%)	132 (75)
**Charact** **eristics of the ** **Careger**		
Caregiver Relationship	Mother, n (%)	137 (78)
	Father, n (%)	22 (12)
	Other, n (%)	17 (10)
Housing	Formal, n (%)	119 (68)
	Informal, n (%)	50 (28)
	Missing, n (%)	7 (4)
Employment	Full-time, n (%)	60 (34)
	Part-time, n (%)	22 (13)
	Unemployed, n (%)	88 (50)
	Missing, n (%)	6 (3)
Highest Level of Education completed	Tertiary, n (%)	35 (20)
	High School, n (%)	89 (51)
	Primary School or Lower, n (%)	46 (26)
	Missing, n (%)	6 (3)
Daily hours spent caring for the child	0–3 h, n (%)	5 (3)
	4–6 h, n (%)	31 (18)
	7–10 h, n (%)	36 (20)
	> 10 h, n (%)	98 (56)
	Missing, n (%)	6 (3)

*Renal Disease includes dialysis and transplantation

#Gastrointestinal Disease includes Liver Disease and/or transplantation

VAS: Visual Analogue Scale; LSS: Level Sum Score

**Table 2:** Ceiling effect for the EQ-TIPS-3L and EQ-TIPS-5L

	Total (*N* = 176)		
	EQ-TIPS-3L	EQ-TIPS-5L	Absolute Difference (percentage points)*
	*n* (%)	*n* (%)	
*Ceiling effect (111111)*	53 (30)	49 (28)	-2
Movement	147 (84)	146 (83)	-1
Play	158 (90)	159 (90)	0
Pain	131 (74)	127 (72)	-2
Social Interaction	153 (87)	154 (88)	+ 1
Communication	150 (85)	153 (87)	+ 2
Eating	126 (72)	130 (74)	+ 2
*Floor effect (333333/555555)*	0 (0)	0 (0)	0
Movement	13 (7)	2 (1)	-6
Play	7 (4)	2 (1)	-3
Pain	8 (5)	0 (0)	-5
Social Interaction	6 (3)	2 (1)	-2
Communication	7 (4)	1 (1)	-3
Eating	11 (6)	4 (2)	-4

*Negative absolute difference reflects a reduction in the ceiling effect on the EQ-TIPS-5L compared to the EQ-TIPS-3L. A positive absolute difference reflects an increase in the ceiling effect on the EQ-TIPS-5L compared to the EQ-TIPS-3L. (*N* = 176)

**Fig. 3:**
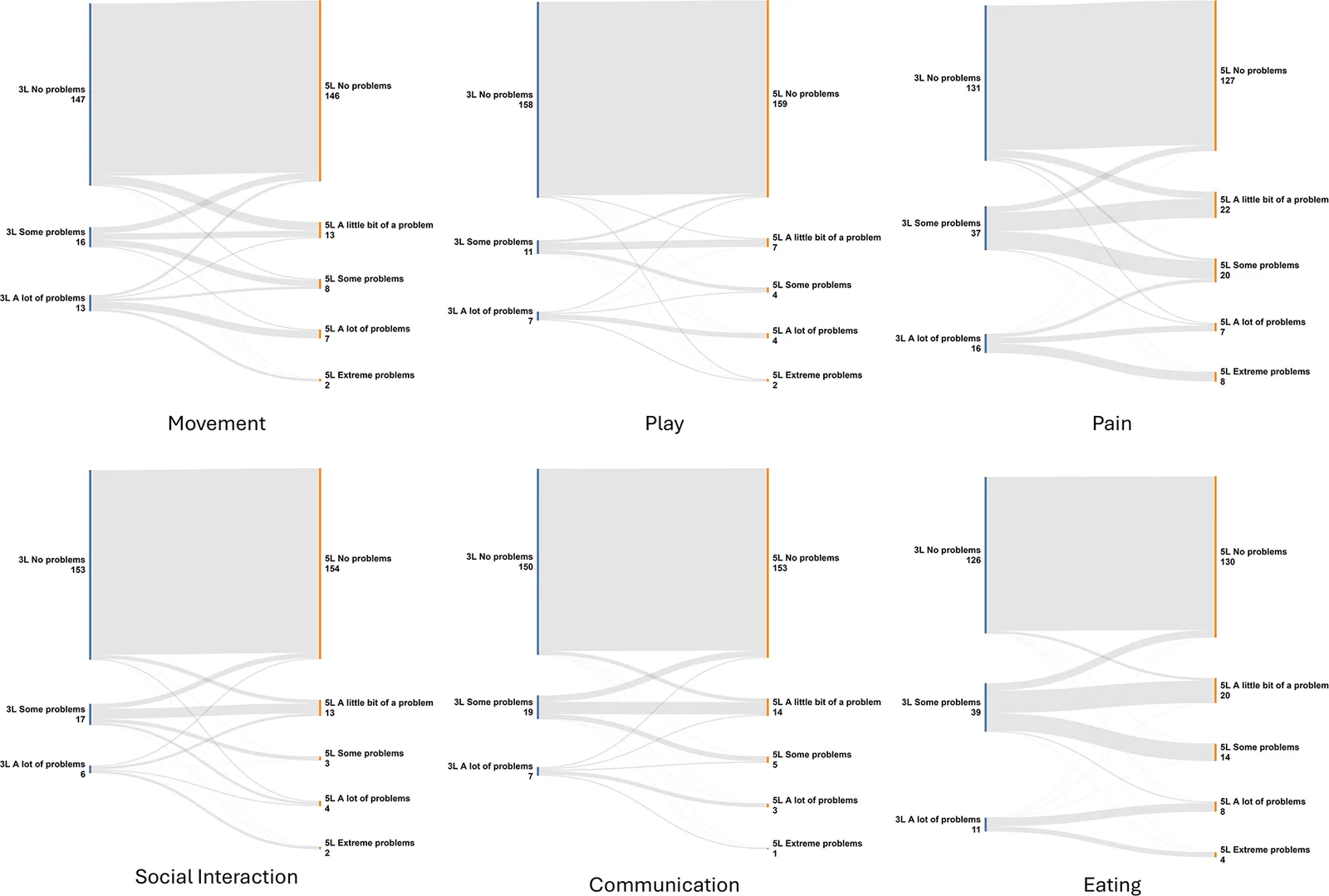
Redistribution of responses: Sankey Plots by dimension

The Sankey Plots in Fig. 3 and redistribution of responses in Supplementary Table S1 show that across all dimensions, the level of reporting “no problems” across dimensions Pain and Eating had a greater spread of problems on the 3L and 5L versions. This high reporting of “no problems” was similarly reported across both the 3L and 5L version with 97.7% to 100% of responses redistributing as anticipated from “no problems” on the 3L to “no problems” or “a little bit of a problem” on the 5L. There were very few responses on Level 2 and 3 on the 3L to redistribute with minimal opportunity for inconsistent responses. Eating had no inconsistent responses. There was a total of 15 (1.4%) inconsistent responses across all dimensions and levels.

For the small number of caregivers who reported the most severe level of the EQ-TIPS-3L (A lot of problems) the majority redistributed to the equivalent level label on the EQ-TIPS-5L, despite it being the 4th severity level and not the 5th (Movement [6/13], Play [4/7], Pain [5/8], Social Interaction [1/6], Communication [3/7], and Eating [4/11] (Supplementary Table S1). There were however a notable number of caregivers whose responses redistributed from "A lot of problems" on the EQ-TIPS-3 L to a more severe level on the EQ-TIPS-5L (extreme problems) across most dimensions except Pain (Movement [2/13], Play [1/7], Pain [0/8], Social Interaction [2/6], Communication [1/7], and Eating [4/11] (Supplementary Table S1). Randomisation was tested as a covariate and found to be non-significant.

The informativity of dimensions (H’ index) was higher across all dimensions on the 5L compared to the 3L, with an average difference across dimensions of 0.096, indicating better informativity (see Table 3**)**. The spread of responses on the 3L and 5L were distributed similarly for all dimensions. The J’ index was expected to remain consistent or minimally changed, and this was largely observed. The spread of responses was greatest for Eating (+ 0.230), indicating that the 5L captures a more even distribution across this dimension. Eating has the highest reporting of problems which allowed distributional variance to populate additional levels. For children where eating is anticipated to be a problem the 5L will be more informative. Interpretation of the H’ and J’ indices for other dimensions was limited due to the low reporting of problems. Randomisation was tested as a covariate and found to be non-significant.

As anticipated, strong correlations were observed between the same dimensions across the two EQ-TIPS versions (see Table 4). Gamma correlations were moderate to strong across all EQ-TIPS dimensions, except for Movement and Communication and Movement and Eating. Randomisation was tested as a covariate, and significant differences were found for both Gamma and Kendall’s Tau B between the 3L and L dimensions. Completing the 3L version first showed a significantly higher correlation between 3L Play and 5L Play (*p* < 0.001). Conversely, completing the 5L version first showed higher correlation between 3L Social Interaction and 5L Social Interaction (*p* = 0.007) and 3L Movement and 5L Play (*p* = 0.004), respectively.

**Fig. 4:**
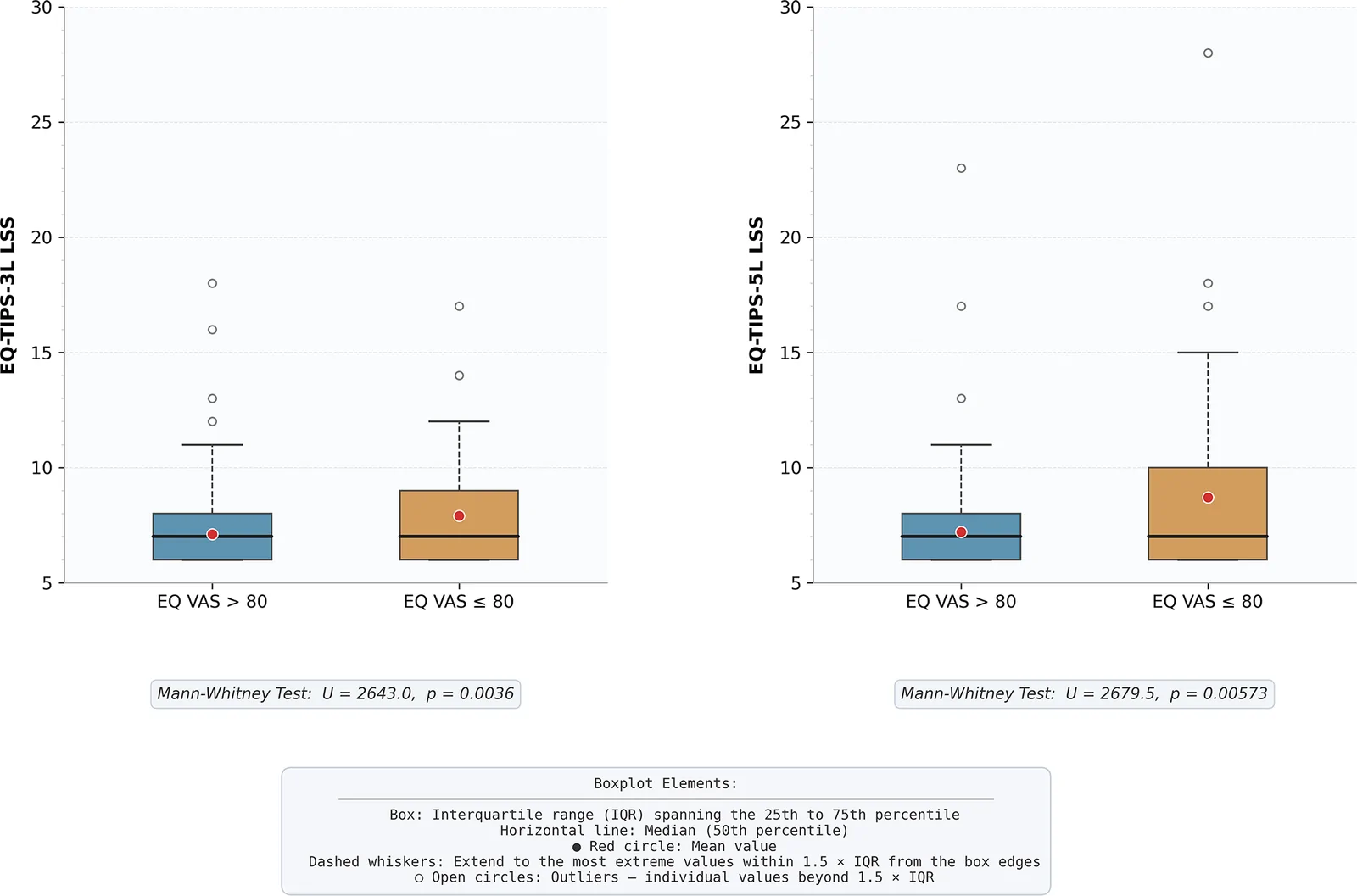
EQ-TIPS-3L and EQ-TIPS-5L LSS by EQ VAS scores

**Fig. 5:**
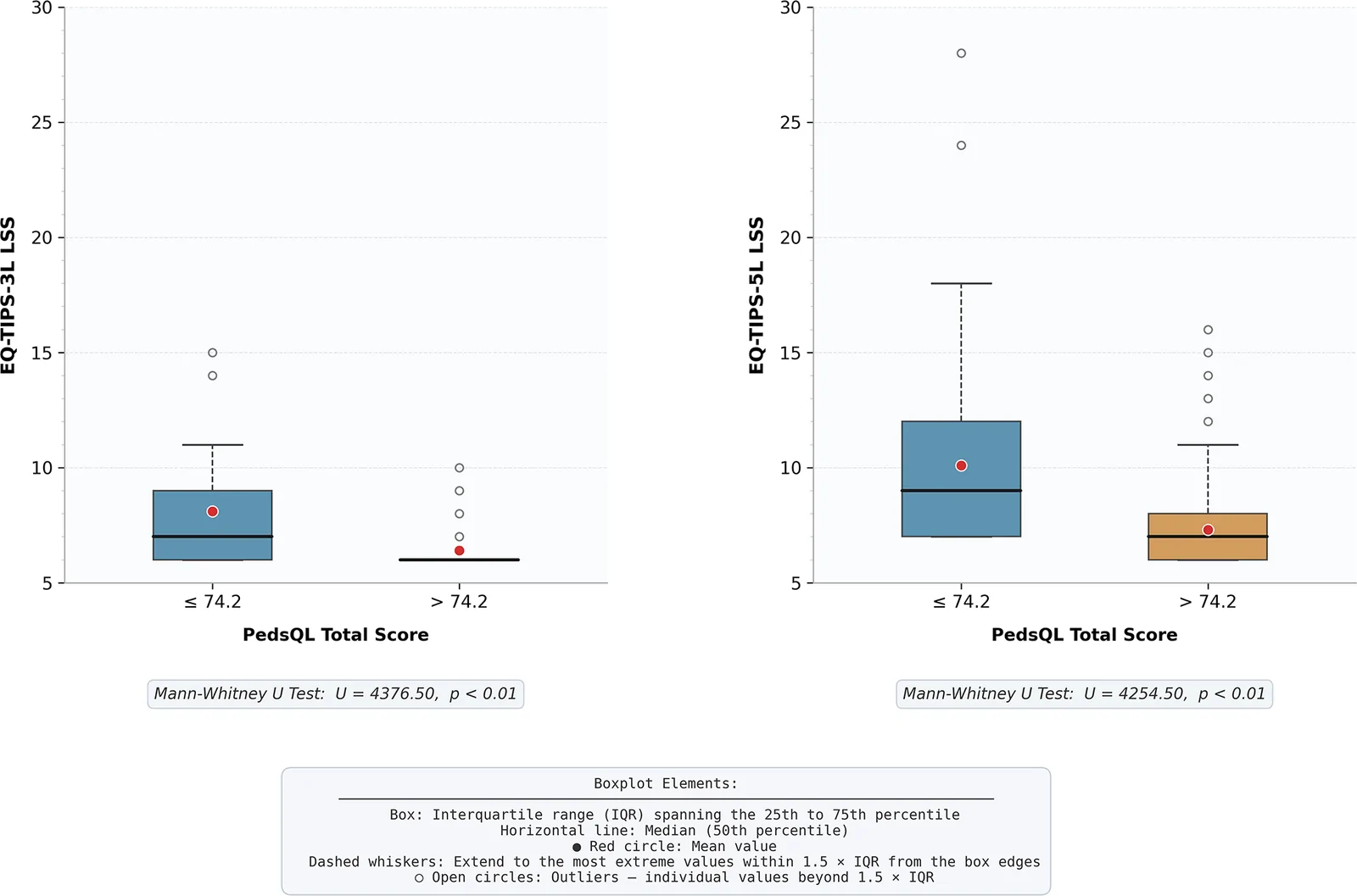
EQ-TIPS-3L and EQ-TIPS-5L LSS by PedsQL Total Scores

Both EQ-TIPS-3L and EQ-TIPS-5L LSS were able to discriminate between known-groups based on the EQ VAS (cut-off 80, Fig. 4) and the PedsQL total scores (cut-off 74.2, Fig. 5). Randomisation was tested as a covariate and found to be non-significant.

**Table 3:** Shannon Index (H′) and Shannon Evenness Index (J′) for the EQ-TIPS-3L and EQ-TIPS-5L dimensions

	EQ-TIPS-5L		EQ-TIPS-3L	
	Shannon Index H’	Shannon Evenness Index J’	Shannon Index H’	Shannon Evenness Index J’
Movement	0.667	0.210	0.561	0.268
Play	0.443	0.269	0.398	0.352
Pain	0.887	0.169	0.688	0.262
Social Interaction	0.516	0.244	0.463	0.328
Communication	0.523	0.147	0.505	0.160
Eating	0.899	0.322	0.746	0.092

**Table 4:** Convergent validity of the EQ-TIPS-3L and EQ-TIPS 5L dimension scores

	Gamma Correlations						Kendall Tau b Correlations					
	EQ-TIPS-5L						EQ-TIPS-5L					
**EQ-TIPS-3L**	Movement	Play	Pain	Social Interaction	Communication	Eating	Movement	Play	Pain	Social Interaction	Communication	Eating
Movement	0.946	0.883	0.722	0.646	0.620	0.357	0.706	0.494	0.410	0.287	0.273	0.154
Play	0.846	0.976	0.827	0.824	0.665	0.618	0.461	0.833	0.432	0.433	0.262	0.270
Pain	0.627	0.813	0.958	0.664	0.411	0.613	0.314	0.388	0.787	0.301	0.158	0.355
Social Interaction	0.611	0.838	0.716	0.960	0.875	0.597	0.268	0.442	0.368	0.758	0.540	0.287
Communication	0.341	0.550	0.535	0.852	0.977	0.595	0.119	0.190	0.246	0.509	0.787	0.282
Eating	0.444	0.607	0.697	0.675	0.735	0.996	0.199	0.244	0.434	0.320	0.365	0.881

## Discussion

This study represents the first evaluation of a potential 5-level version of the EQ-TIPS, an important undertaking given the instrument’s reliance solely on proxy responses to assess young children’s HRQoL. This reliance raises unique challenges, as the intrinsic and extrinsic influences on proxy responses when rating HRQoL in this age group remain unclear.

The health conditions of this sample are heterogeneous but arguably include severe conditions, with many diagnosed with a haematological/oncological disease. The perceived general health, as measured on the EQ VAS, was only slightly higher (Median 90) when compared to older paediatric patients, 8–17 years, with similar haematological malignancies in China (Median 84) [40]. Despite similar general health scores, there were far more problems reported on the EQ-5D-Y-3L and EQ-5D-Y-5L than on the EQ-TIPS versions. For dimensions with obvious conceptual overlap, the EQ-5D-Y-5L [40] had relatively fewer reports of “no problems”; for Mobility (60%), Usual Activities (60%) and Pain/Discomfort (54%) compared to the EQ-TIPS-5L dimensions of Movement (83%), Play (90%) and Pain (72%). There was, however, a similar low reduction ceiling effect, total and by dimension, when moving from the 3-level version to the 5-level version of the EQ-TIPS (range 0–4% points), to previous studies comparing the 3-level and 5-level versions of the EQ-5D-Y in haematological malignancies in China (range 0–5% points) [40], orthopaedic conditions in South Africa (range 0–6% points) [41] and healthy and sick children in Malawi (range 0–5% points) [42]. Further evaluation of psychometric performance between the EQ-TIPS-3L and 5L will further strengthen the evidence base.

The ceiling effect, or reporting of no problems, observed on the EQ-TIPS-3L in this study was similarly reported in children with chronic conditions in Australia [20] (ranging from 61.8% for Eating to 88% for Movement, and 43% for 111111), Afrikaans for South Africa [43] (ranging from 77% for Eating to 93% for Communication, and 46% for 111111), and isiXhosa for South Africa (ranging from 77% for Eating to 92% for Communication, and 50% for 111111). This trend in reporting of no problems was lower in infants and toddlers with acute illness, including COVID [40] (ranging from 57% for Eating to 74% for Movement, and 30% for 111111) and RSV [44] (ranging from 10% for Pain to 30% for Movement).

An analysis of EQ-TIPS dimensions with the lowest reported problems and their association with developmental milestones reveals strong correlations, particularly between Movement, Play, Social Interaction and Communication [45]. This underscores the interconnectedness of these domains in early childhood. In acute illness, the primary focus is often on treating the immediate condition. Once stabilised, attention can shift towards addressing other developmental areas. However, in chronic conditions like epilepsy, which can significantly impact development, concerns may persist. The concept of the “disability paradox” or response shift to a new normal becomes relevant, as children and families adapt to the challenges posed by the condition [46]. This phenomenon suggests that parents or caregivers may report fewer problems than expected, potentially due to adjusted expectations and a shifted frame of reference. For children with chronic conditions that affect development, the point of reference for normalcy may also shift. The family or the healthcare setting may become the new benchmark for comparison. This altered perception of normal may influence reporting across all developmental domains, except for changes in areas such as Eating or Pain, which are more likely to be discussed and addressed in healthcare settings. Self-reports on the EQ-5D-Y-3L suggest that at least for physical dimensions, there is maintenance of this response shift, notably in children with physical impairments into older age [47, 48].

When examining comparable dimensions across these instruments, a moderate-to-strong correlation is expected [6]. The order of randomisation, however, affected these correlations for Play, Social Interaction, and Movement, which may be attributed to a learning effect or to the influence of the other outcome measures completed between the two EQ-TIPS versions. In infants and toddlers with chronic conditions, problems with Movement, Play, Social Interaction, and Communication may not differ significantly from those of their peers, the reference case in the EQ-TIPS. This is likely due to the high dependence on caregivers at this early age [6], which can buffer the impact of developmental delays or challenges. As children grow older and gain more independence, the developmental disparities may become more pronounced. The influence of referencing age-appropriate behaviour or developmental norms needs further investigation in qualitative research. The effect of age on the reporting of problems by dimension should be explored with subgroup analysis by age and/or in longitudinal research.

The influence of proxy reporting of health states remains challenging. Because infants and toddlers depend on their caregivers, independent assessment of HRQoL may be impossible [6]. This may be particularly pertinent in this study setting, South Africa. South Africa is an Upper Middle-Income Country with massive inequality reflected in our high Gini Index [49]. For many parts of the country, resources are limited, with low incomes, high unemployment, high crime rates, and poor living conditions, including high levels of overcrowding and reliance on informal housing, and restricted access to running water and/or electricity [50]. These circumstances result in a lower educational level of mothers, lower socio-economic status of the families, and psychosocial stress in the families. Moreover, this results in increased vulnerability to disease, which is often exacerbated by health inequalities [51]. The sample of caregivers in this study aligns with the general population, with the majority having basic education or less and moderate levels of unemployment. This sample mirrors the socioeconomic context typical of many African or low-income communities. This demographic profile ensures comparability with previous EQ-5D-Y-3L and EQ-5D-Y-5L data collected within similar settings. However, these factors may also influence HRQoL reporting, as communities are well-versed in ill health and socioeconomic stressors. The authors postulate that this may result in reduced recognition of the consequences of poor health. The association between poor health literacy, notably the ability to understand written information and confidence in completing written forms, and poor health outcomes is well established in adult health care, with most data reported from High-Income Countries [52–55]. How this affects reporting in Low- and Middle-Income Countries, and how it affects reporting on health outcomes for a young child, are currently unclear. It is unclear whether this poor understanding is limited to health severity or extends to the EQ-TIPS dimension headings. Furthermore, having the survey available only in English may have limited the comprehension of the questionnaire. Although only around 7% of South Africans speak English as their home language, it is widely taught in schools [56, 57]. Nearly all primary school education shifts from African languages to English as the language of learning and teaching by Grade 4 and is the preferred language of instruction by most [56, 57]. It is thus not surprising that caregivers felt comfortable completing the survey in English. It is, however, well recognised that education in the home language is more effective, and that teaching and learning in other languages, especially in settings with limited resources and support, may pose challenges to proficiency [56]. Testing the EQ-TIPS across different geographical, cultural, and socio-economic contexts is recommended to provide further insight into proxy reporting and to support the instrument’s validity.

### Study limitations

The cross-sectional design of this study did not allow for full exploration of performance; the potential superior performance of the 5-level version in responsiveness and test-retest reliability testing over time warrants further investigation to inform recommendations on the use of the EQ-TIPS versions. Furthermore, the small subgroup sizes for several conditions and known groups limited condition-specific inferences and known-group analysis. The use of an English instrument in a multilingual setting may have further limited the interpretation of these results. The high ceiling across EQ-TIPS dimensions on the 3L and 5L versions limits the interpretation of Shannon’s indices. Targeted recruitment of infants and toddlers with conditions likely to lead to greater variation in EQ-TIPS problem reporting is warranted. To enhance the granularity of future data analysis, it is recommended to stratify by age group and disease severity. This approach may reveal nuanced patterns and associations that are not readily apparent in aggregated data and inform future recommendations for the instrument’s use.

## Conclusion

These results indicate that a 5L version of the EQ-TIPS EV2.0 does not influence the ceiling effect in the same way as the EuroQol Adult and Youth descriptive system. However, for children expected to have eating problems, the 5L version offers greater informativeness. Similarly, for health conditions where more severe problems with movement, pain, and eating are anticipated, the 5L allows more severe reporting with reduced floor effects. The modest increase in informativity of the EQ-TIPS-5L indicates the potential advantages of the expanded levels of reporting, but this requires testing in samples with more frequent reporting of severe health states. The pattern of reporting in this sample may be due to the nature of the included health conditions or to proxy reporting in toddlers and infants, where dependence on caregivers remains notable. Responsiveness testing of the 3L and 5L studies should be prioritised in future work. Furthermore, future development of the EQ-TIPS should include qualitative evidence from diverse geographical and cultural contexts to better understand whether the wording of the dimension headings may be contributing to the lack of sensitivity between the 3L and 5L versions.

## Supplementary Information

Below is the link to the electronic supplementary material.

Supplementary Material 1

## Data Availability

All data is available from the author on reasonable request.

## References

[1] Sachs JD (2012) From millennium development goals to sustainable development goals. Lancet 379:2206–2211. 10.1016/S0140-6736(12)60685-0 22682467

[2] Sharrow D, Hug L, You D et al (2022) Global, regional, and national trends in under-5 mortality between 1990 and 2019 with scenario-based projections until 2030: a systematic analysis by the UN Inter-agency Group for Child Mortality Estimation. Lancet Glob Health 10:e195–e206. 10.1016/S2214-109X(21)00515-5 PMC8789561 35063111

[3] Irwin LG, Siddiqi A, Hertzman C (2007) Early child development: a powerful equalizer

[4] Viholainen H, Ahonen T, Cantell M et al (2016) The Early Motor Milestones in Infancy and Later Motor Skills in Toddlers. Phys Occup Ther Pediatr 26:91–113. 10.1080/J006v26n01 16938827

[5] Dietrich KN, Eskenazi B, Schantz S et al (2005) Principles and Practices of Neurodevelopmental Assessment in Children: Lessons learned from the Centers for Children’s Environmental Health and Disease Prevention Research. Environ Health Perspect 113:1437–1446. 10.1289/ehp.7672 PMC1281293 16203260

[6] Oliveira C, de Silva NT, Ungar WJ et al (2020) Health-related quality of life in neonates and infants: a conceptual framework. Qual Life Res 29:1159–1168. 10.1007/s11136-020-02432-6 31997081

[7] Azzopardi PS, Kerr JA, Francis KL et al (2023) The unfinished agenda of communicable diseases among children and adolescents before the COVID-19 pandemic, 1990–2019: a systematic analysis of the Global Burden of Disease Study 2019. Lancet 402:313–335. 10.1016/S0140-6736(23)00860-7 PMC10375221 37393924

[8] UNICEF (2025) Children with disabilities. In: UNICEF DATA. https://www.unicef.org/health/childhood-diseases. Accessed 15 Feb 2025

[9] Kwon J, Smith S, Raghunandan R et al (2023) Systematic Review of the Psychometric Performance of Generic Childhood Multi-attribute Utility Instruments. Appl Health Econ Health Policy 21:559–584. 10.1007/s40258-023-00806-8 PMC10232649 37133712

[10] Jabrayilov R, van Asselt ADI, Vermeulen KM et al (2018) A descriptive system for the Infant health-related Quality of life Instrument (IQI): Measuring health with a mobile app. PLoS ONE 13:1–14. 10.1371/journal.pone.0203276 PMC6118381 30169510

[11] Furlong W, Rae C, Feeny D et al (2023) Preference-Based Assessments Generic Health-Related Quality of Life Utility Measure for Preschool Children (Health Utilities Preschool): Design, Development, and Properties. Value Health 26:251–260. 10.1016/j.jval.2022.07.015 36031479

[12] Saigal S, Rosenbaum P, Stoskopf B et al (2005) Development, reliability and validity of a new measure of overall health for pre-school children. Qual of 14:243–257 10.1007/s11136-004-4228-7 15789958

[13] Verstraete J, Ramma L, Jelsma J (2020) Item generation for a proxy health related quality of life measure in very young children. Health Qual Life Outcomes 18:11. 10.1186/s12955-020-1271-1 PMC6961344 31937311

[14] Verstraete J, Ramma L, Jelsma J (2020) Validity and reliability testing of the Toddler and Infant (TANDI) Health Related Quality of Life instrument for very young children. J Patient Rep Outcomes 4. 10.1186/s41687-020-00251-4 PMC7652950 33165662

[15] Varni JW, Limbers Ca, Neighbors K et al (2011) The PedsQL™ Infant Scales: feasibility, internal consistency reliability, and validity in healthy and ill infants. Qual Life Res 20:45–55. 10.1007/s11136-010-9730-5 20730626

[16] Varni JW (2015) PedsQL Measurement Model for the Pediatric Quality of Life Inventory. https://www.pedsql.org 10.1097/00005650-199902000-00003 10024117

[17] DeLuca KE, Dalziel K, Wittenberg E et al (2024) Selecting PedsQL items to derive the PedsUtil health state classification system to measure health utilities in children. Health Qual Life Outcomes 22:1–14. 10.1186/s12955-024-02268-5 PMC11238509 38987772

[18] Kwon J, Raghunandan R, Nghiem SH et al (2024) Development of a Health-State Classification System for the Pediatric Quality-of-Life Inventory Version 4.0 Generic Core Scales for Preference-Based Valuation in Australia. Value Health. 10.1016/j.jval.2024.08.005 39343089

[19] Verstraete J, Lloyd AJ, Jelsma J (2021) Performance of the Toddler and Infant (TANDI) Health-Related Quality of Life Instrument in 3-4-Year-old Children. Children 8:1–15. 10.3390/children8100920 PMC8534352 34682184

[20] Pan T, Huang L, He V et al (2025) Comparison of the psychometric performance of experimental EuroQol Toddler and Infant Populations Instrument (EQ-TIPS) and Pediatric Quality of Life Inventory™ (PedsQL) in young children. Eur J Health Econ. 10.1007/s10198-025-01804-4 PMC13046681 40736640

[21] Devlin NJ, Xie F, Slaap B, Stolk E (2025) Measuring and Valuing Health Using EuroQol Instruments: New Developments 2025 and Beyond. Appl Health Econ Health Policy. 10.1007/s40258-025-00989-2 PMC12535937 40705271

[22] Lamb A, Murray A, Lovett R (2021) The challenges of measuring and valuing quality of life in preschool children: a retrospective review of NICE appraisals. Children 8 10.3390/children8090765 PMC8464668 34572196

[23] Rivero-Arias O, Png ME, White A et al (2024) Benefits and harms of antenatal and newborn screening programmes in health economic assessments: the VALENTIA systematic review and qualitative investigation. Health Technol Assess (Rockv) 28. 10.3310/PYTK6591 PMC11228689 38938110

[24] Kennedy-Martin M, Slaap B, Herdman M et al (2020) Which multi-attribute utility instruments are recommended for use in cost-utility analysis? A review of national health technology assessment (HTA) guidelines. Eur J Health Econ 21:1245–1257. 10.1007/s10198-020-01195-8 PMC7561556 32514643

[25] EuroQol Research Foundation (2024) EQ-5D-Y-5L User Guide

[26] Kreimeier S, Åström M, Burström K et al (2019) EQ-5D-Y-5L: developing a revised EQ-5D-Y with increased response categories. Qual Life Res 0:0. 10.1007/s11136-019-02115-x PMC6571085 30739287

[27] Herdman M, Gudex C, Lloyd A et al (2011) Development and preliminary testing of the new five-level version of EQ-5D (EQ-5D-5L). Qual Life Res 20:1727–1736. 10.1007/s11136-011-9903-x PMC3220807 21479777

[28] EuroQol Research Foundation (2025) EQ-TIPS (Toddler and Infant Populations). In: https://euroqol.org/research-at-euroqol/eq-tips/

[29] Devlin N, Parkin D, Janssen B (2020) Methods for analysing and reporting EQ-5D data 33347096

[30] Verstraete J, Booth C, Booth J et al (2023) Fighting to breathe and fighting for health-related quality of life: measuring the impact of being dependent on technology for breathing on the child and their caregiver. Patient. 10.1007/s40271-023-00657-4 PMC10769921 37991685

[31] Harris PA, Taylor R, Minor BL et al (2019) The REDCap consortium: Building an international community of software platform partners. J Biomed Inf 95 10.1016/j.jbi.2019.103208 PMC7254481 31078660

[32] Janssen MF, Birnie E, Haagsma JA, Bonsel GJ (2008) Comparing the standard EQ-5D three-level system with a five-level version. Value Health 11:275–284. 10.1111/j.1524-4733.2007.00230.x 18380640

[33] Pickard AS, Leon MC, De, Kohlmann T et al (2007) Psychometric Comparison of the Standard EQ-5D to a 5 Level Version in Cancer Patients. Med Care 45:259–263 10.1097/01.mlr.0000254515.63841.81 17304084

[34] Janssen MF, Birnie E, Bonsel GJ (2007) Evaluating the discriminatory power of EQ-5D, HUI2 and HUI3 in a US general population survey using Shannon’s indices. Qual Life Res 16:895–904. 10.1007/s11136-006-9160-6 PMC1915610 17294285

[35] Cohen J (1968) Weighted kappa: Nominal scale agreement provision for scaled disagreement or partial credit. Psychol Bull 70:213–220 10.1037/h0026256 19673146

[36] Varni JW, Burwinkle TM, Seid M, Skarr D (2003) The PedsQL 4.0 as a pediatric population health measure: feasibility, reliability, and validity. Ambul Pediatr 3:329–341 10.1367/1539-4409(2003)003<0329:tpaapp>2.0.co;2 14616041

[37] Chen HY, Cisler RA (2011) Assessing health-related quality of life among children with special health care needs in the United States. Children’s Health Care 40:311–325. 10.1080/02739615.2011.617239

[38] Jones R, Mulhern B, McGregor K et al (2021) Psychometric performance of HRQoL measures: an australian multi-instrument comparison study protocol (P-MIC). Children 8 10.3390/children8080714 PMC8393323 34438605

[39] Jones R, O’Loughlin R, Xiong X et al (2023) Comparative psychometric performance of common generic paediatric health-related quality of life instrument descriptive systems: results from the Australian paediatric multi-instrument comparison study. Pharmacoeconomics. 10.1007/s40273-023-01330-2 PMC11169028 37955799

[40] Zhou W, Li Y, Busschbach J et al (2024) Psychometric validation of the Chinese versions of EQ-5D-Y-3L and the experimental EQ-TIPS in children and adolescents with COVID-19. The European Journal of Health Economics 10.1007/s10198-024-01710-1 PMC11937211 39066840

[41] Verstraete J, Marthinus Z, Dix-Peek S, Scott D (2022) Measurement properties and responsiveness of the EQ-5D-Y-5L compared to the EQ-5D-Y-3L in children and adolescents receiving acute orthopaedic care. Health Qual Life Outcomes 20:10.1186/s12955-022-01938-6RESEARCH PMC8851798 35177084

[42] Ngwira LG, Maheswaran H, Verstraete J et al (2023) Psychometric performance of the Chichewa versions of the EQ-5D-Y and EQ-5D-Y-5L among healthy and sick children and adolescents in Malawi. J Patient Rep Outcomes 7. 10.1186/s41687-023-00560-4 PMC9996597 36892714

[43] Verstraete J, Amien R (2023) Cross-Cultural Adaptation and Validation of the EuroQol Toddler and Infant Populations (EQ-TIPS) instrument into Afrikaans for South Africa. Value Health Reg Issues 35:78–86. 10.1016/j.vhri.2023.01.009 36905789

[44] Taborda I, Oben G, Santos Ferreira C et al (2025) Loss of Health-related quality of life among hospitalized children with respiratory syncytial virus and their caregivers. Pediatric Inf Dis J. 10.1097/INF.0000000000004973 40924757

[45] Verstraete J, Amien R, Herdien L (2024) EuroQol Toddler and Infant Populations (EQ-TIPS): Age-Related Differences in Performance. Children 11:1–13. 10.3390/children11081034 PMC11353094 39201968

[46] Barclay-Goddard R, King J, Dubouloz CJ, Schwartz CE (2012) Building on transformative learning and response shift theory to investigate health-related quality of life changes over time in individuals with chronic health conditions and disability. Arch Phys Med Rehabil 93:214–220. 10.1016/j.apmr.2011.09.010 22289229

[47] Scott D, Ferguson GD, Jelsma J (2017) The use of the EQ-5D-Y health related quality of life outcome measure in children in the Western Cape, South Africa: psychometric properties, feasibility and usefulness - a longitudinal, analytical study. Health Qual Life Outcomes 1–14. 10.1186/s12955-017-0590-3 PMC5248508 28103872

[48] Scott D, Scott C, Jelsma J, Abraham D, Verstraete J (2019) Validity and feasibility of the self-report EQ-5D-Y generic health-related quality of life outcome measure in children and adolescents with Juvenile Idiopathic Arthritis in Western Cape, South Africa. South Afr J Physiotherapy 75 10.4102/sajp.v75i1.1335 PMC6676980 31392295

[49] World Bank Group (2025) Gini Index - South Africa. In: https://data.worldbank.org/indicator/SI.POV.GINI?locations=ZA

[50] Richards R, O’Leary B, Mutsonziwa K (2007) Measuring quality of life in informal settlements in South Africa. Soc Indic Res 81:375–388. 10.1007/s11205-006-9014-1

[51] Shortt NK, Hammett D (2013) Housing and health in an informal settlement upgrade in Cape Town, South Africa. J Housing Built Environ 28:615–627. 10.1007/s10901-013-9347-4

[52] Al Sayah F, Qiu W, Johnson JA (2016) Health literacy and health-related quality of life in adults with type 2 diabetes: a longitudinal study. Qual Life Res 25:1487–1494. 10.1007/s11136-015-1184-3 26603739

[53] Katz P, Dall’Era M, Trupin L et al (2021) Impact of Limited Health Literacy on Patient-Reported Outcomes in Systemic Lupus Erythematosus. Arthritis Care Res (Hoboken) 73:110–119. 10.1002/acr.24361 PMC7775267 32741118

[54] Tormey LK, Reich J, Chen YS et al (2019) Limited health literacy is associated with worse patient-reported outcomes in inflammatory bowel disease. Inflamm Bowel Dis 25:204–212. 10.1093/ibd/izy237 29992302

[55] Hahn EA, Burns JL, Jacobs EA et al (2015) Health Literacy and Patient-Reported Outcomes: A Cross-Sectional Study of Underserved English-and Spanish-Speaking Patients with Type 2 Diabetes. J Health Commun 20:4–15. 10.1080/10810730.2015.1061071 26513026

[56] Milton C, du Plessis S, van der Heever H (2020) English as an additional language: Professional development needs of early childhood practitioners in historically disadvantaged contexts. South Afr J Child Educ 10:1–10. 10.4102/sajce.v10i1.804

[57] Spaull N (2016) Disentangling the language effect in South African schools: Measuring the impact of language of assessment in grade 3 literacy and numeracy. South Afr J Child Educ 1:1–20. 10.4102/sajce

