# Supplementary material for: Expanding the experimental EuroQol toddler and infant populations (EQ-TIPS) descriptive system: comparing 3-level and 5-level performance for infants and toddlers with chronic conditions
Source: J Patient Rep Outcomes. 2026 Jun 12;10:203. doi: 10.1186/s41687-026-01113-1 (PMC13739895; doi:10.1186/s41687-026-01113-1)
Supplement: Supplementary file 1 [file 41687_2026_1113_MOESM1_ESM.docx]

Supplementary Table S1. Redistribution and inconsistency between the EQ-TIPS-3L and EQ-TIPS-5L dimension responses

| **EQ-TIPS-3L** | **EQ-TIPS-5L** | | | | |  |  |
| --- | --- | --- | --- | --- | --- | --- | --- |
| **Movement** | No | A little bit | Some | A lot | Extreme | Total | Inconsistencies, n () |
| No | 139 | 7 | 1 | 0 | 0 | 147 | 1 (1) |
| Some | 5 | 5 | 5 | 1 | 0 | 16 | 0 |
| A lot | 2 | 1 | 2 | 6 | 2 | 13 | 3 (23) |
| Total | 146 | 13 | 8 | 7 | 2 | 176 | 4 (2) |
| Inconsistencies, n () | 2 (1) | 1 (8) | 1 (13) | 0 | 0 | 4 (2) |  |
| **Play** |  |  |  |  |  |  |  |
| No | 156 | 1 | 0 | 0 | 1 | 158 | 1 (1) |
| Some | 2 | 6 | 3 | 0 | 0 | 11 | 0 |
| A lot | 1 | 0 | 1 | 4 | 1 | 7 | 1 (14) |
| Total | 159 | 7 | 4 | 4 | 2 | 176 | 2 (1) |
| Inconsistencies, n () | 1 (1) | 0 | 0 | 0 | 1 (50) | 2 (1) |  |
| **Pain** |  |  |  |  |  |  |  |
| No | 122 | 6 | 2 | 1 | 0 | 131 | 3 (2) |
| Some | 5 | 16 | 15 | 1 | 0 | 37 | 0 |
| A lot | 0 | 0 | 3 | 5 | 0 | 8 | 0 |
| Total | 122 | 6 | 2 | 1 | 0 | 176 | 3 (2) |
| Inconsistencies, n () | 0 | 0 | 2 (100) | 1 (100) | 0 | 3 (2) |  |
| **Social Interaction** |  |  |  |  |  |  |  |
| No | 149 | 3 | 0 | 1 | 0 | 153 | 1 (1) |
| Some | 4 | 8 | 3 | 2 | 0 | 17 | 0 |
| A lot | 1 | 2 | 0 | 1 | 2 | 6 | 3 (50) |
| Total | 154 | 13 | 3 | 4 | 2 | 176 | 4 (2) |
| Inconsistencies, n () | 1 (1) | 2 (15) | 0 | 1 (25) | 0 | 4 (2) |  |
| **Communication** |  |  |  |  |  |  |  |
| No | 147 | 3 | 0 | 0 | 0 | 150 | 0 |
| Some | 5 | 10 | 4 | 0 | 0 | 19 | 0 |
| A lot | 1 | 1 | 1 | 3 | 1 | 7 | 2 (29) |
| Total | 153 | 14 | 4 | 3 | 1 | 176 | 2 (1) |
| Inconsistencies, n () | 1 (1) | 1 (7) | 0 | 0 | 0 | 2 (1) |  |
| **Eating** |  |  |  |  |  |  |  |
| No | 124 | 2 | 0 | 0 | 0 | 126 | 0 |
| Some | 6 | 18 | 14 | 1 | 0 | 39 | 0 |
| A lot | 0 | 0 | 0 | 7 | 4 | 11 | 0 |
| Total | 130 | 20 | 14 | 8 | 4 | 176 | 0 |
| Inconsistencies, n () | 0 | 0 | 0 | 0 | 0 | 0 |  |

Shaded cells indicate inconsistency
